# *PLoS Neglected Tropical Diseases*: Two Years of Providing Access to Innovation for the World's Poor … and Counting

**DOI:** 10.1371/journal.pntd.0000494

**Published:** 2009-07-28

**Authors:** Peter J. Hotez, Serap Aksoy

**Affiliations:** 1 Sabin Vaccine Institute and Department of Microbiology, Immunology, and Tropical Medicine, George Washington University Medical Center, Washington, D.C., United States of America; 2 Division of Epidemiology of Microbial Diseases, Yale School of Public Health, New Haven, Connecticut, United States of America

It has been almost two years since *PLoS Neglected Tropical Diseases* was launched as the first open-access journal devoted to the neglected tropical diseases (NTDs). At that time we aspired to achieve multiple goals by creating a new journal for the NTD community. We believed that through an open-access format, we would provide an immediate and vital mechanism by which the community of NTD scientists, public health experts, and health care professionals would have access to the latest information about these conditions. Back in 2007, we were especially excited about reaching investigators in the disease-endemic countries, particularly those who often cannot afford the expense of some of the more traditional and established biomedical journals. To help realize these goals we went to great lengths to secure a high level of editorial board representation from disease-endemic countries in Latin America, Africa, and Asia. We also hoped that through our frontmatter articles that included viewpoints, commentaries, reviews, and editorials, *PLoS Neglected Tropical Diseases* would have an important role in advocacy and elevating the profile of the NTDs.

Based on a number of metrics that we have been following, it is clear that our community journal is moving in the right direction and in some cases has already achieved some of its goals. Article submissions to the journal have been steadily increasing from throughout the world, with roughly one-half from “the North,” i.e., the United States, Canada, and Europe (and also Australia), and the other half from “the South,” i.e. Latin America and the Caribbean, Africa, and Asia. Among the countries in the North we anticipated receiving a large number of article submissions from the United States and the United Kingdom, and indeed this has been the case. But we were pleasantly surprised to observe that France is tied with the United Kingdom for the third largest number of article submissions. We have also received a large number of article submissions from Switzerland, Australia, Germany, Belgium, Canada, The Netherlands, Sweden, and Spain. In the coming year we hope to see an increase in submissions from Japan and the Republic of Korea, where we know there is a tremendous amount of high-quality scientific activity on the NTDs.

The observation that almost one-half of our article submissions come from the NTD-endemic countries is especially gratifying. Brazil now accounts for the second largest number of article submissions, behind the United States. Submissions from Brazil have been extremely high in quality and cover a wide range of topics, from molecular pathogenesis and clinical aspects to epidemiology and policy. There is also wide representation from a diversity of research institutes (including FIOCRUZ, the Oswaldo Cruz Foundation) and universities. One of us recently had the opportunity to attend Med Trop 2009 (the annual Brazilian Congress of Tropical Medicine, held this year in Recife) in order to thank the community there for their support and interest. We are also excited by the large number of article submissions from elsewhere in Latin America, including Peru, Mexico, and Argentina.

We continue to receive a large number of article submissions from sub-Saharan Africa, especially from Kenya, Uganda, Tanzania, Ethiopia, Côte d'Ivoire, Burkina Faso, Cameroon, Ghana, Sudan, and Niger. Because of the enormous disease burden in Africa and the importance of building capacity there, we want to continue to promote scientific activities in this region and encourage submissions. Therefore we look forward to continue hearing from you in this regard. The number of submissions from Nigeria and South Africa is much lower than we had hoped for, given the high quality of scientific activities that we understand are in place in these countries. Clearly this is something we must work on in the coming months. We hope to receive more papers from the Middle East—including both Israel and Iran. The large number of article submissions from Asia—specifically India and China—is gratifying, but we are striving to increase submissions to a level similar to that from Brazil. Submissions from Bangladesh and Pakistan need to increase, as well as those from Southeast Asia, especially Indonesia. We are pleased about the large number of articles that we receive from Thailand, Vietnam, and Lao DR.

In addition to the quantity of papers, the quality of papers published in *PLoS Neglected Tropical Diseases* has remained high. A relatively small percentage of all submitted articles are accepted for publication, and we believe that this high quality is reflected in our first impact factor of 4.2 (based on the last nine weeks of 2007 after the journal was first launched), which positions the journal as a leader in the area of tropical medicine. Both the print and electronic media have been unusually receptive to reporting on scientific results published in *PLoS Neglected Tropical Diseases*. Information about our scientific findings and frontmatter pieces has appeared frequently and regularly in general scientific and medical journals such as *Science*, *Nature*, and *The Lancet*, as well as high-profile lay publications such as the *New York Times*, *Los Angeles Times*, *Reuters*, *AP*, *USA Today*, *Wall Street Journal*, *Financial Times*, and *The Economist*, among others. We are grateful to the journalists who cover NTDs for these publications, as they provide an important role in not only informing readers about these conditions, but also serving to elevate the profile of these diseases. We feel that this intensive media coverage has also provided an important advocacy role in getting NTDs onto the agendas of a number of international agencies, such as the United Nations, the World Health Organization, and the governments representing the Group of Eight (G8) countries, including some nations that have provided substantial donor support. We are also very grateful to the Bill & Melinda Gates Foundation for providing early seed funding for launching our journal.

Most of the NTDs have been well represented in *PLoS Neglected Tropical Diseases* during its first two years, including, of course, helminth and protozoan infections, but also many bacterial and viral (especially arboviral) infections. The numerous submissions we have received on bacterial and viral NTDs (and their vectors) has motivated us to expand our editorial board in order to add expertise on these conditions. Indeed, the overwhelming response from the NTD community with respect to the number and quality of article submissions has prompted us to revisit how we accommodate and manage growth. We are therefore pleased to announce that former Deputy Editor Serap Aksoy from Yale University has joined Peter Hotez as Editor-in-Chief of the journal. Serap is a leading authority on the vector biology of human African trypanosomiasis, but she also has wide-ranging interests on all aspects of microbial pathogens and their vectors. She has been instrumental in capacity-building for research in endemic countries. Currently she either manages or assists training programs in China, Colombia, Turkey, Uganda, and Kenya in the area of vector borne disease control. She also chairs the WHO/TDR committee on Innovative Vector Control. In 2008 in Kampala, Uganda, Serap led an interactive (and highly attended) writing workshop on behalf of *PLoS Neglected Tropical Diseases*. Serap grew up in Turkey and completed her education in the United States at Vassar College and then Columbia University. We have known each other for more than 20 years, having first worked closely together as postdoctoral fellows in the laboratory of Professor Frank Richards at Yale University. Together with the outstanding editorial staff at PLoS, we look forward to jointly promoting journal activities and outreach and working closely with the Editorial Board. These last two years have been exciting ones for not only *PLoS Neglected Tropical Diseases*, but indeed for the entire NTD community, and we look forward to hearing from you in the coming weeks and months.

